# Genome-Wide Analysis of Gene Families of Pattern Recognition Receptors in Fig Wasps (Hymenoptera, Chalcidoidea)

**DOI:** 10.3390/genes12121952

**Published:** 2021-12-05

**Authors:** Hong-Xia Hou, Da-Wei Huang, Zhao-Zhe Xin, Jin-Hua Xiao

**Affiliations:** Institute of Entomology, College of Life Sciences, Nankai University, Tianjin 300071, China; 1120180393@mail.nankai.edu.cn (H.-X.H.); huangdw@nankai.edu.cn (D.-W.H.); 1120180392@mail.nankai.edu.cn (Z.-Z.X.)

**Keywords:** pattern recognition receptor, innate immunity, adaptive evolution, fig wasp

## Abstract

Pattern recognition receptors (PRRs) play important roles in detecting pathogens and initiating the innate immune response. Different evolutionary histories of pollinators and non-pollinators may result in different immune recognition systems. A previous study had reported that there were significant differences in peptidoglycan recognition proteins (PGRPs) between pollinators and non-pollinators in gene number and lineage of specific genes. In this study, based on the genomic data of 12 fig wasp species, with seven pollinators and five non-pollinators, we investigated the evolution patterns of PRRs, such as Gram-negative bacteria-binding proteins (GNBPs), C-type lectins (CTLs), scavenger receptors class B (SCRBs), fibrinogen-related proteins (FREPs), galectins, and thioester-containing proteins (TEPs). Our results showed that pollinators had no GNBP, but non-pollinators all had two gene members, which were clustered into two different clades in the phylogenetic tree, with each clade having specific domain and motif characteristics. The analysis of CTL and SCRB gene families also showed that there were lineage-specific genes and specific expansion in non-pollinators. Our results showed that there were significant differences in immune recognition between pollinators and non-pollinators, and we concluded that they had undergone flexible adaptive evolution in different environments. Our study can provide more molecular evidence for future functional studies on the immune system of fig wasps.

## 1. Introduction

Immune recognition is the first step of an effective immune response to protect hosts from invading pathogens [1]. When pathogens break through the physical barrier and enter the hemocoel of hosts, they will encounter immune recognition receptors and trigger humoral and cellular immune responses in the hosts [2,3]. The pattern recognition receptors (PRRs) of the hosts play important roles in recognizing pathogens by sensing the pathogen-associated molecular patterns (PAMPs) of microbes, such as peptidoglycan, lipopolysaccharide, lipoteichoic acid, and β-1,3-glucans [4]. The PRRs of insects are diverse, including peptidoglycan recognition proteins (PGRPs), Gram-negative bacteria-binding proteins (GNBPs), C-type lectins (CTLs), galectins, scavenger receptors (SRs), thioester-containing proteins (TEPs), and fibrinogen-related proteins (FREPs) etc. These PRRs have their own characteristics and functions, and accurately identify non-self entities, playing important roles in innate immunity [2]. Different microorganisms would selectively activate different immune recognition genes, indicating a flexible innate immune system to cope with various bacterial infections [5].

Most immune recognition genes exist in the form of gene families, which have undergone birth and death evolution [6]. New genes were created by duplication, some of which have been retained for a long time, while some were lost because of the accumulation of harmful mutations. Diverse immune genes contribute to dealing with different pathogenic infections [7]. Gene duplication represents the core process of multigene family evolution, while pseudogene formation, gene loss, recombination and natural selection also show effects in shaping the evolution of gene families to varying degrees [8]. Compared with the fruit fly *Drosophila melanogaster*, the number of immune recognition genes of the house fly *Musca domestica* is significantly expanded, such as Nimrods and TEPs, which help to increase the ability to resist the infection of pathogens from the complex environment [9]. Eleven GNBPs were identified in the genome of *Daphnia pulex*, and phylogenetic analysis showed that there was a species-specific expansion of 10 GNBP paralogues, many of which were clustered in the same scaffold, implying that the GNBPs of *D. pulex* probably arose from local duplication events [10]. The GNBPs in the American cockroach *Periplaneta americana* and black soldier fly *Hermetia illucens* have also been expanded, with 12 and 14 gene members respectively, closely related to the diversity of pathogens in the living environment [11,12]. However, during their long period of evolution, the immune genes of some insects present gene contraction or gene loss. For example, there is only one PGRP gene in the human body louse (*Pediculus humanus*), significantly less than *D. melanogaster* (13 genes), *Bombyx mori* (12 genes), and *Anopheles gambiae* (seven genes) [13]. Amazingly, the pea aphid *Acyrthosiphon pisum* appears to have no PGRPs [14]. The significant decrease or lack of PGRPs in the human body louse and pea aphid are probably associated with their diets with fewer microbes.

Fig wasps (Hymenoptera, Chalcidoidea) live in the compact fig syconia of fig trees, and rely on fig syconia during their whole life cycle, including for habitat and food sources. Based on whether they pollinate the fig trees, fig wasps can be classified into pollinating fig wasps (pollinators) and non-pollinating fig wasps (non-pollinators) [15]. Fig syconia provide stable and suitable place for fig wasps to live, and pollinators pollinate the figs. Thus, the pollinators have a symbiotic relationship with the figs. However, most non-pollinators do not pollinate the figs, and likely have a parasitic relationship. Phylogenetic analysis in previous studies showed that the association of pollinators and non-pollinators with figs have evolved at least twice independently [16]. Therefore, there are significant differences between pollinators and non-pollinators. For example, the lifestyles of pollinators and non-pollinators are different, especially in the aspects of diets and spawning styles. The pollinators are herbivorous, and complete the progress of development, mating and oviposition in the fig syconia. However, the non-pollinators have complex diets, acting as gallmakers, inquilines, or parasites. When they mature, they fly out of the fig syconia, and oviposit outside of the syconia. Although both pollinators and non-pollinators have close relationships with the figs, the differences in lifestyles between them lead to different exposure patterns to pathogens. As a model for mutualistic symbiosis in their evolution, fig syconia provide a relatively closed environment for pollinators to lead a more stable life than non-pollinators, preventing various bacterial infections. Previous studies of fig wasps’ innate immunity have identified the gene members of immune signal pathways, as well as some pathogen receptors, such as peptidoglycan recognition proteins (PGRPs). The results showed that pollinators possessed a more streamlined immune system than non-pollinators [17,18]. Since those studies are enlightening, there is a need to explore the differences in the other immune recognition receptors between pollinators and non-pollinators.

As natural materials, the fig wasps cannot be bred in artificial environment, so they provide us with an excellent opportunity to study immune genes in the natural environment. In this study, based on the genome data of 12 fig wasp species with seven pollinators (*Eupristina koningsbergeri*, *Kradibia gibbosae*, *Platyscapa corneri*, *Wiebesia pumilae*, *Dolichoris vasculosae*, *Ceratosolen solmsi* and *Ceratosolen fusciceps*) and five non-pollinators (*Sycophaga agraensis*, *Sycobia* sp.2, *Philotrypesis tridentata*, *Apocrypta bakeri* and *Sycophila* sp.2) (Table S1), we focused on the differences in immune recognition receptors between pollinators and non-pollinators. The 12 fig wasps we used were from several figs, and we collected as many species as possible to illustrate our results. We identified putative PRRs, including GNBPs, CTLs, SCRBs, FREPs, galectins, TEPs. We studied the evolutionary relationships with orthologs from other insects, and analyzed the specificity of pollinators or non-pollinators. Our results will provide more genome evidence concerning the adaptive evolution of innate immunity of insects.

## 2. Materials and Methods

### 2.1. Gene Identification and Feature Prediction

The gene identification was mainly based on the genomes of 12 fig wasps. The genome sequences of fig wasps in our study were submitted to NCBI (project accession PRJNA277475, PRJNA641212 and PRJNA494992). Blast orthologs and HMMER prediction were both used to identify the gene members of immune recognition families. Firstly, the PRR gene sequences of *D. melanogaster*, *Apis mellifera*, *Nasonia vitripennis* and *Pteromalus puparum* were regarded as queries, and local blast was performed with an *E*-value of 10^−5^ to search for putative immune recognition genes in fig wasp species’ genomes. Meanwhile, based on the model of query genes in the Pfam database, HMMER was used to predict putative immune recognition genes from fig wasp genomes. Then, the NCBI conserved domain database (https://www.ncbi.nlm.nih.gov/Structure/cdd/wrpsb.cgi, accessed on 27 September 2021) was used to predict the domains of putative genes to ensure the accuracy of genes. Finally, the combination of the results of the two methods were considered to be a comprehensive result. SignalP-5.0 Server (http://www.cbs.dtu.dk/services/SignalP/, accessed on 8 October 2021) was used to predict the signal peptide. TMHMM Server v. 2.0 (http://www.cbs.dtu.dk/services/TMHMM/, accessed on 8 October 2021) was used to predict the transmembrane domain. EXpasy (https://web.expasy.org/compute_pi/, accessed on 15 October 2021) was used to predict molecule weight and theoretical isoelectric point.

### 2.2. Phylogenetic Analysis

The phylogenetic tree was constructed with all the genes from 12 fig wasp species (Table S1) and four reference species, *D. melanogaster*, *A. mellifera*, *N. vitripennis* and *P. puparum*. The entire amino acid dataset of genes was aligned using MAFFT v7.037 with default settings [19]. IQ-TREE v1.6.12 was used to predict the best fit model and construct the phylogenetic tree with the maximum likelihood (ML) method [20]. The results of phylogenetic trees were visualized by FigTree v1.4.3. The Interactive Tree of Life (iTOL) (http://itol.embl.de/, accessed on 20 October 2021) was used to polish the phylogenetic trees.

### 2.3. Gene Family Expansion and Contraction

CAFE v4.2.1 was employed to estimate gene family expansion and contraction with the default parameters [21]. A divergence time tree was used, which was constructed with 12 fig wasp species and five other species, *N. vitripennis*, *A. mellifera*, *D. melanogaster*, *A. pisum*, and *D. pulex*.

### 2.4. Functional Divergence Analysis

Diverge v3.0 software was used to investigate the functional divergence of gene families [22,23]. Type I functional divergence represents amino acid patterns, the sites of which show great variation between clusters. Based on different evolutionary rates, the clades show different functions.

### 2.5. Genomic Location Analysis

TBtools v1.085 software was used to analyze the genomic locations and tandem duplication of the PRR genes [24]. Genomic protein sequences and genome annotation documents were used to perform the analysis.

## 3. Results

### 3.1. Gram-Negative Bacteria-Binding Proteins (GNBPs)

**Figure 1:**
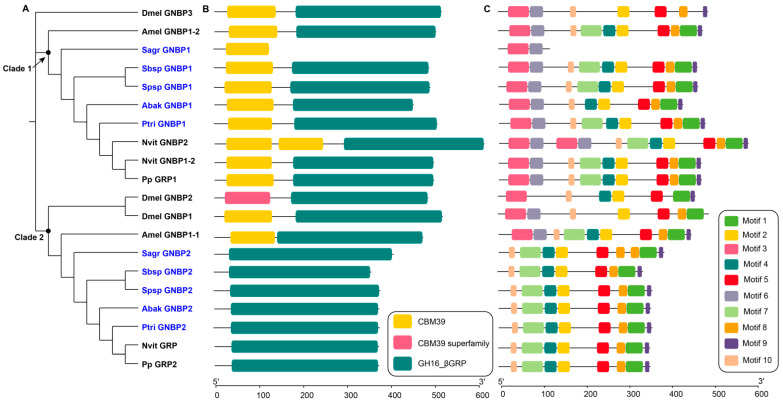
Phylogenetic relationships (**A**), conserved domains (**B**) and motifs (**C**) analysis of GNBPs. The amino acid sequences of GNBPs of five non-pollinators and *D. melanogaster* (Dmel), *A. mellifera* (Amel), *N. vitripennis* (Nvit), *P. puparum* (Pp) were used to construct the maximum likelihood tree, and the optimal model was LG+I+G4. The non-pollinators were marked with blue, and the four reference species were marked with black.

GNBPs are a family that show high affinity for β-1,3-glucan, which are numerous in fungal cell walls. GNBPs play important roles in innate immune recognition, initiation of the Toll pathway and melanization. Interestingly, in our study, we found no GNBP genes in pollinator genomes, and two GNBP genes in every non-pollinator genome (Table 1). The GNBPs of non-pollinators were clustered into two clades in the phylogenetic tree (Figure 1A). In clade 1, Sagr_GNBP1 only contained one N-terminal domain, which might participate in carbohydrate recognition, and the other genes contained one N-terminal domain and one C-terminal domain (Figure 1B). The different domains contained specific motif characteristics (Figure 1C). The amino acid sequence lengths of GNBPs of the fig wasps in clade 1 mostly ranged from 429 to 482. In contrast, the amino acid sequence length of Sagr_GNBP1 was only 119 (Table S2). In clade 2, the GNBPs of five non-pollinators, *N. vitripennis* and *P. puparum* contained only one C-terminal domain, while the other GNBPs contained two domains (Figure 1B). The amino acid sequence lengths of GNBPs of the fig wasps in clade 2 ranged from 339 to 386, most of which lacked transmembrane regions (Table S2). The C-terminal-β-1,3-glucanase-like domains in non-pollinators lacked putative catalytic sites, which was consistent with other insects, such as *D. melanogaster* and *A. mellifera* (Figure S1).

### 3.2. C-Type Lectins (CTLs)

**Figure 2:**
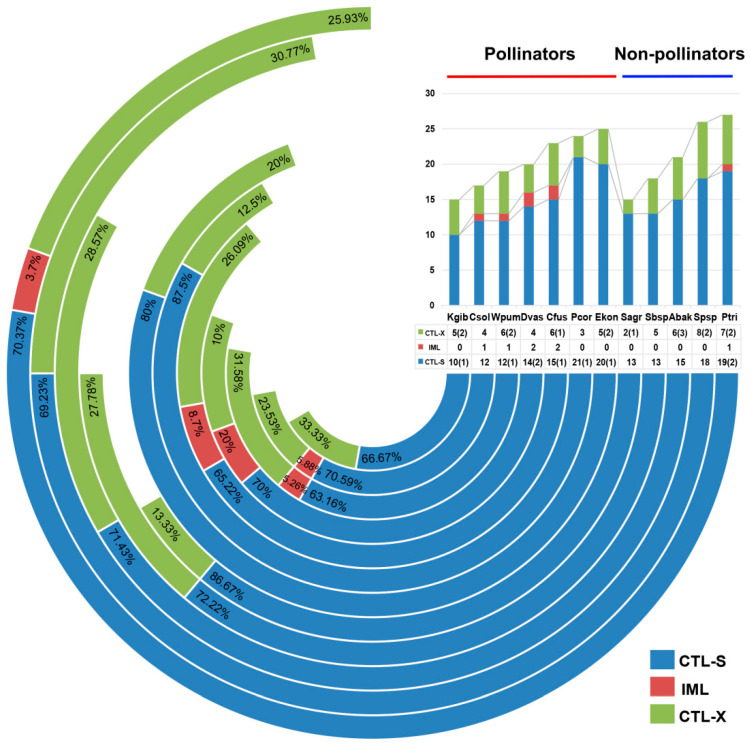
Statistical analysis of CTL types in the 12 fig wasp species. CTLs were divided into CTL-S, IML and CTL-X based on the domain characteristics. CTL-S (blue) contained only one or three CTL domains, IML (red) contained two CTL domains, and CTL-X (green) contained one or more CTL domains and additional domains. The column diagram on the top right showed the percentage of CTL-S, IML and CTL-X of 12 fig wasps, and the corresponding numbers were listed in the table below the diagram. The number in parentheses of CTL-S represented the genes all containing three CTL domains, and that of CTL-X represented the genes containing two or three CTL domains.

CTLs are a family of lectins widely distributed in metazoans, containing carbohydrate-recognition domains (CRD) to mediate ligand binding [25,26]. The CRD domains of CTLs contain highly conserved amino acid residues that specifically bind to sugars. The conserved glutamic-proline-asparagine (EPN) motif in the sequence of CTLs specifically binds to mannose, and the glutamine-proline-aspartic (QPD) motif specifically binds to galactose [27]. CTLs are highly abundant in many insect genomes with diverse functions. In this study, we identified the gene family members of CTLs in the fig wasps. The number of gene members varied from species to species, but they were more numerous than in *A. mellifera*, while fewer than in *D. melanogaster* (Table 1). There was no significant difference in the number of CTLs between pollinators and non-pollinators. Two non-pollinator species of *P. tridentata* and *Sycophila* sp.2 possessed the most CTLs, but the pollinator species of *K. gibbosae* and a non-pollinator species of *S. agraensis* had the fewest CTLs. According to the characteristics of domains, there are three types: CTL-S (only one CRD), IML (two tandem CRDs), CTL-X (CRD and other additional domains). In fig wasps, CTL-S was the most common type, followed by CTL-X (Figure 2). The domain architecture analysis suggested that the CTLs showed a stronger ability to bind galactose than mannose, because there were more CTLs containing a QPD motif (Table 2).

Phylogenetic analysis suggested that there were some conserved clades and some lineage-specific clades among fig wasps. For example, clade 2 and clade 9-1 were strict 1:1:1 orthologs of the 12 fig wasp species and the four reference species. Clade 16 contained homologous genes of all fig wasps and the four reference species, but *E. koningsbergeri* and *N. vitripennis* both had two gene members from gene expansion. Clade 7 contained the homologous genes of all non-pollinators and only one pollinator *K. gibbosae*, probably suggesting a lineage-specific gene of non-pollinators. Clade 12 and clade 13 both showed lineage-specific gene expansion of non-pollinators (Figure S2). There were some gene groups clustered together with closed relationships in the phylogenetic tree, such as the groups marked in red of *P. corneri*, *E. koningsbergeri*, *C. fusciceps*, *Sycobia* sp.2, and *S. agraensis* (Figure S2). We analyzed the genomic locations of CTLs on the genomes of the 12 fig wasps, and found that most of the gene groups gathered in the phylogenetic tree were located in the same scaffold as tandem genes (Figure S3).

**Figure 3:**
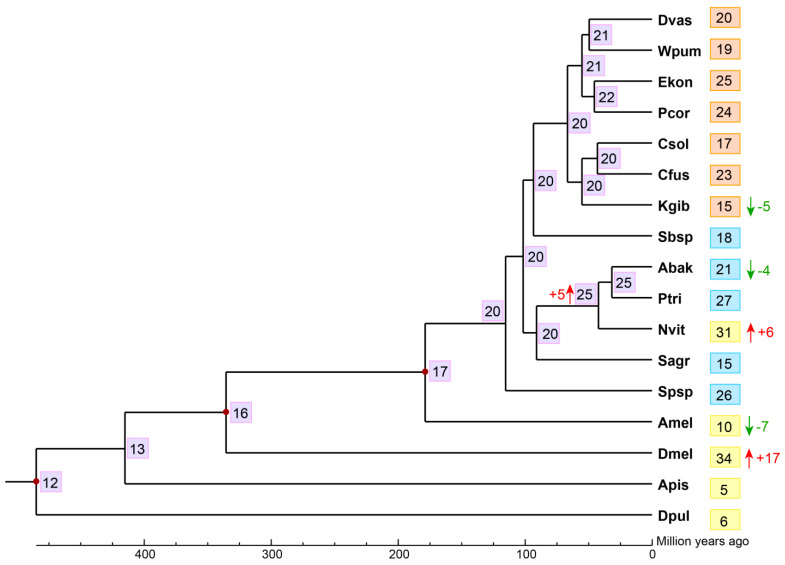
Analysis of gene expansion and contraction of CTLs of fig wasps. The number in the gray background on each node represented the estimated copy number of ancestral CTLs. Dvas, *D. vasculosae*; Wpum, *W. pumilae*; Ekon, *E. koningsbergeri*; Pcor, *P. corneri*; Csol, *C. solmsi*; Cfus, *C. fusciceps*; Kgib, *K. gibbosae*; Sbsp, *Sycobia* sp.2; Abak, *A. bakeri*; Ptri, *P. tridentata*; Sagr, *S. agraensis*; Spsp, *Sycophila* sp.2; Dmel, *D. melanogaster*, Amel, *A. mellifera*; Nvit, *N. vitripennis*; Apis, *A. pisum*; Dpul, *D. pulex*. The numbers in the orange background represented the gene number of CTLs in the corresponding pollinators. The numbers in the blue background represented the gene number of CTLs in non-pollinators. The numbers in the yellow background indicated the number of CTL genes in the corresponding reference species. The red upward arrows indicated gene expansion (*E*-value < 0.05), and the green downward arrows indicated gene contraction (*E*-value < 0.05).

We estimated the gene expansion and contraction of CTL gene family in the 12 fig wasps using CAFE, finding that the most recent common ancestor of the Chalcidoidea likely had approximately 20 CTLs. There was a net loss (*E*-value < 0.05) of five CTLs during the evolution of *K. gibbosae* from its common ancestor with *C. solmsi* and *C. fusciceps*; there was also a net loss (*E*-value < 0.05) of four CTLs during the evolution of *A. bakeri* from its common ancestor with *P. tridentata*, even though there was a net expansion (*E*-value < 0.05) of five CTLs in their common ancestor with *N. vitripennis* (Figure 3).

### 3.3. Scavenger Receptor B (SCRBs)

**Figure 4:**
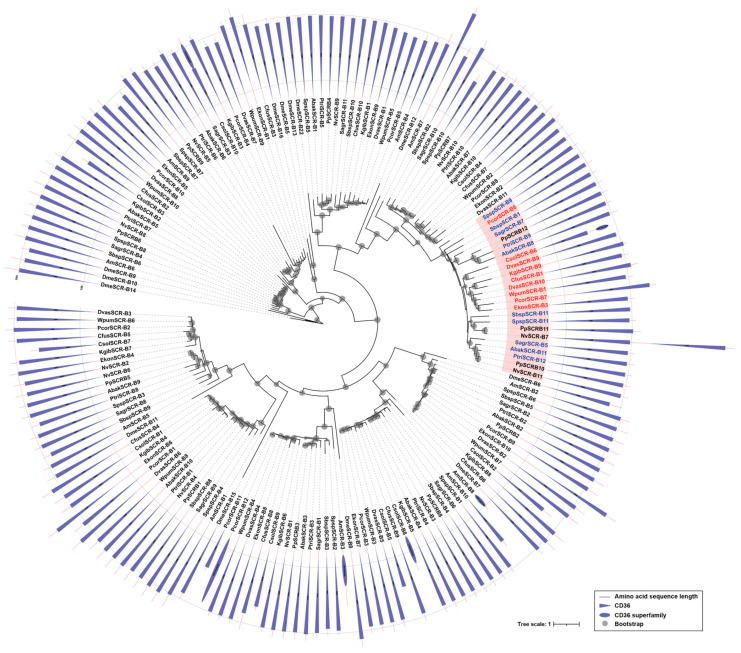
Phylogenetic analysis of SCRBs. The amino acid sequences of SCRBs of 12 fig wasp species (Table S1), *D. melanogaster* (Dme), *A. mellifera* (Am), *N. vitripennis* (Nv), *P. puparum* (Pp) were used to construct the maximum likelihood tree, and the optimal model was LG+F+R8. The duplicated genes in non-pollinators were filled in pink background, the pollinators were presented in red, and the non-pollinators were presented in blue. Domains of SCRBs were showed in the outer ring. CD36 was represented by a triangle, and the CD36 superfamily was represented by an oval.

Scavenger receptors class B are a type of scavenger receptors, playing vital roles in pathogen clearance and maintenance of homeostasis [28]. In this study, the gene numbers of SCRBs in fig wasps ranged from 10 to 12, while the non-pollinators all had 11 SCRBs (Table 1). In pollinators, most species had 10 SCRBs, but *P. corneri* had the most (12 SCRBs), followed by *D. vasculosae* (11 SCRBs). Phylogenetic analysis suggested that there were various conserved orthologs between pollinators and non-pollinators, as well as *N. vitripennis* and *P. puparum*. Interestingly, non-pollinators showed specific expansion (the pink clade in the phylogenetic tree), and expanded genes located in the same scaffold (Figure 4, Figure S4). All the SCRBs had no signal peptide, and a majority of SCRBs contained at least one CD36 domain and two transmembrane regions (Table S2). Two SCRBs (CsolSCR-B6, CsolSCR-B10) in *C. solmsi* and one SCRB (NvSCR-B7) in *N. vitripennis* contained two tandem CD36 domains (Figure 4). 

### 3.4. Fibrinogen-Related Proteins (FREPs)

**Figure 5:**
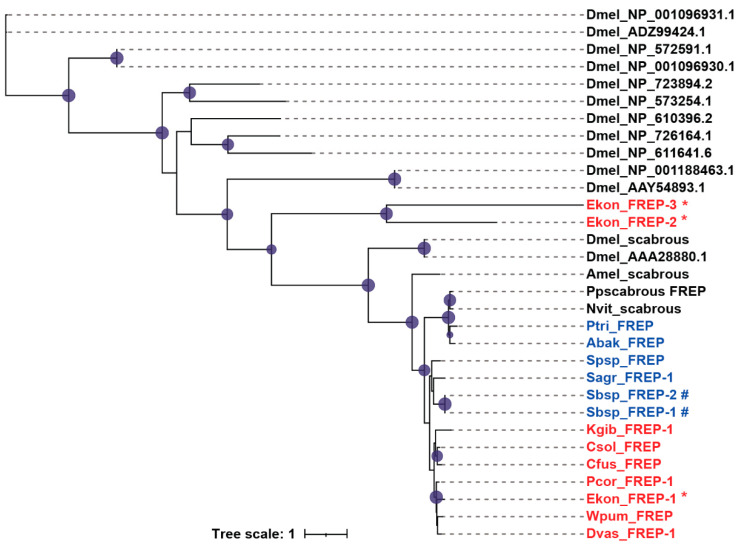
Phylogenetic analysis of FREPs. The amino acid sequences of FREPs of 12 fig wasp species (Table S1), *D. melanogaster* (Dmel), *A. mellifera* (Amel), *N. vitripennis* (Nvit), and *P. puparum* (Pp) were used to construct the maximum likelihood tree, and the optimal model was JTT+I+G4. The pollinators were presented in red, and the non-pollinators were presented in blue. The expanded genes in *E. koningsbergeri* were marked with *, and the expanded genes in *Sycobia* sp.2 were marked with #.

Fibrinogen-related proteins (FREPs) are immune-related proteins that contain fibrinogen domains in the C-terminal region. In invertebrates, FREPs mainly play roles in defending against pathogens [29,30]. We found that the FREP gene numbers in fig wasps were significantly less than that in *D. melanogaster*. Most fig wasp species contained only one FREP; however, the pollinator *E. koningsbergeri* and the non-pollinator *Sycobia* sp.2 showed gene expansion (Table 1). Most FREPs contained a signal peptide in the N-terminal region, except for Ekon_FREP-3, Kgib_FREP-1, Sbsp_FREP-1 and Sbsp_FREP-2 (Table S2). In *E. koningsbergeri* and *Sycobia* sp.2, the two expanded FREPs were clustered into one clade respectively, some of which only contained several motifs (Figure 5, Figure S5).

### 3.5. Galectins

**Figure 6:**
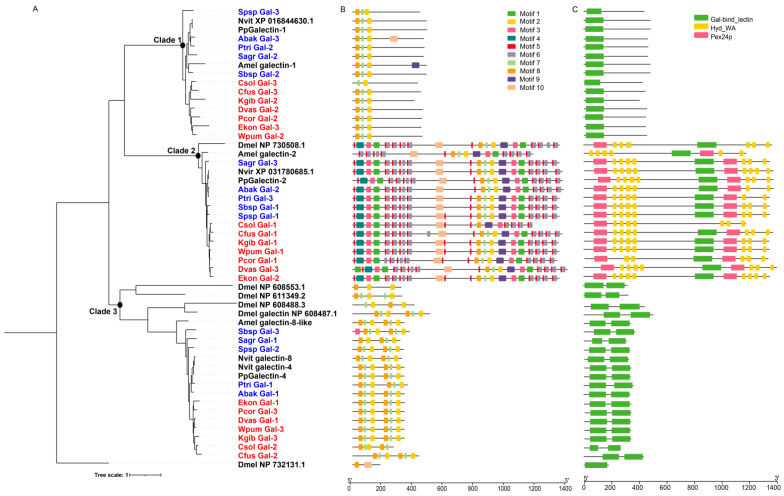
Phylogenetic relationships, conserved domains and motifs analysis of galectins of 12 fig wasps. (**A**) The amino acid sequences of galectins of 12 fig wasp species (Table S1), *D. melanogaster* (Dmel), *A. mellifera* (Amel), *N. vitripennis* (Nvit) and *P. puparum* (Pp) were used to construct the maximum likelihood tree, and the optimal model was JTT+R9. The galectins were clustered into three clades. (**B**) The distributions of conserved motif in galectin genes. 10 motifs were displayed in different colored boxes. (**C**) The domains of galectin genes. Each clade had specific domain features.

Galectins are an evolutionarily-conserved lectin family that bind specifically to β-galactosides, which contain at least one conserved carbohydrate recognition domain (CRD). In *Drosophila*, galectins are involved in innate immune response and development [31]. We identified three galectin genes from each species of the 12 fig wasps, fewer than *D. melanogaster* (6 genes), all of which lacked signal peptides (Table 1 and Figure S2). The galectin genes of fig wasps were clustered into three clades in the phylogenetic tree, suggesting diverse functions (Figure 6A). We analyzed the characteristics of galectin genes and found that each clade presented specific domain and motif characteristics (Figure 6B,C). The galectins of clade 1 contained a single CRD, the molecular weight of which ranged from 401 to 481; the galectins of clade 2 contained one CRD and other domains, the molecular weight of which ranged from 1172 to 1399; and the galectins of the clade 3 contained two tandem CRDs, the molecular weight of which ranged from 265 to 431. Correspondingly, each clade had a specific motif characteristic. Phylogenetic analysis indicated that the galectin genes were conserved in fig wasps, because each clade contained only one galectin gene from each fig wasp species, and one or two genes from *N. vitripennis*, *P. puparum*, and *A. mellifera*. Functional divergence analysis showed that there was significant functional divergence between different clades (*p*-value < 0.01), likely suggesting that the three clades had gone through different genetic evolutions (Table 3). 

### 3.6. Thioester-Containing Proteins (TEPs)

**Figure 7:**
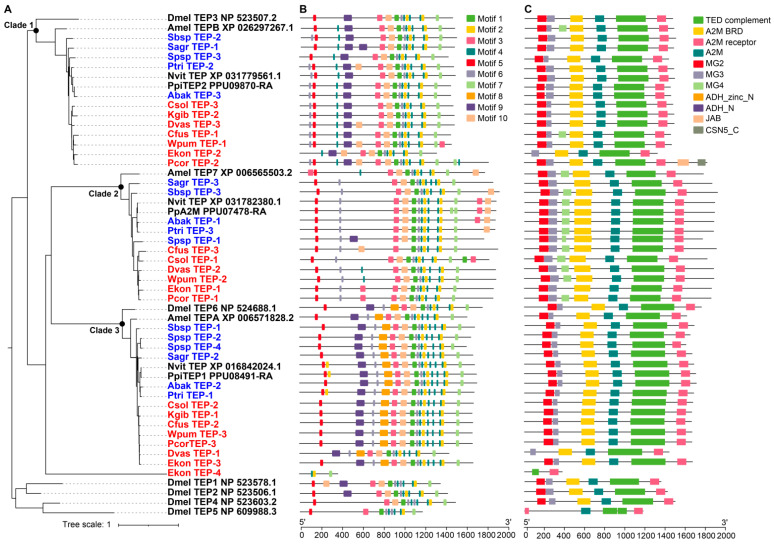
Phylogenetic relationships, conserved domains and motifs analysis of TEPs of 12 fig wasps. (**A**) The phylogenetic tree was constructed using the amino acid sequences of TEPs of 12 fig wasp species, *D. melanogaster* (Dmel), *A. mellifera* (Amel), *N. vitripennis* (Nvit) and *P. puparum* (Pp), and the optimal model was JTTDCMut+F+R5. The TEPs were clustered into three clades. (**B**) The distributions of conserved motif in TEP genes. 10 motifs were displayed in different colored boxes. (**C**) The domains of TEP genes. Each clade had specific domain features.

Thioester-containing proteins (TEPs) are widely distributed in vertebrates and invertebrates, and are involved in recognizing pathogens. Most TEPs contain a thioester motif (GCGEQ) and a catalytic histidine residue, which allow the TEPs to label the pathogen surface with a covalent bond and present to phagocytes to promote phagocytosis and clearance. The number of TEP gene family members of the 12 fig wasp species ranged from two to four, fewer than *D. melanogaster* (6 TEPs) (Table 1). The TEP genes of fig wasps were clustered into three clades in the phylogenetic tree, suggesting their diverse functions (Figure 7A). The domain and motif characteristics of TEPs of each clade were displayed in Figure 7B,C. *K. gibbosae* only had two TEPs, located in clade 1 and clade 3; however, *Sycophila* sp.2 had four TEPs, showing gene expansion in clade 3. The molecular weight of the TEPs of clade 1 ranged from 1317 to 1815, and that of clade 2 and clade 3 ranged from 1767 to 1918 and 1436 to 1706 respectively. Phylogenetic analysis indicated that the TEP genes of *N. vitripennis*, *P. puparum*, and *A. mellifera* were most homologous to those of fig wasps in each clade. Functional divergence analysis showed that there was significant functional divergence between different clades (*p*-value < 0.01), likely suggesting that the three clades had gone through different genetic evolutions (Table 4). 

## 4. Discussion

Pattern recognition receptors play important roles in recognizing pathogens, functioning in the first step of innate immune response. In this study, we identified and analyzed the gene families of GNBP, CTL, SCRB, FREP, Galectin, and TEP of the 12 fig wasp species. Among these recognition proteins, GNBP, CTL, SCRB gene families showed differences between pollinators and non-pollinators, suggesting they had gone through different evolutionary patterns under different backgrounds. The FREP gene family showed gene expansion in *E. koningsbergeri* and *Sycobia* sp.2, which indicated a flexible immune system response to microbes in the environment. Insects in different environments might possess different gene patterns of immune recognition genes to function in innate immunity. There was no PGRP in the *D. pulex* genome, but the expansion of other recognition genes, GNBPs (11 GNBPs), might compensate for the loss of PGRP [10]. In the diamondback moth, *Plutella xylostella*, there were 18 GNBPs, which were clustered into three clades in the phylogenetic tree, indicating a dramatic expansion and diverse functions in effective defense against Gram-negative bacteria and fungi [32]. In *D. melanogaster*, the three GNBPs possess diverse functions, GNBP1 being involved in the initiation of Toll pathway, and GNBP3 related to the recognition of fungi [2]. Interestingly, in our study, we found that there was no GNBP gene in pollinators, but two gene members in every non-pollinator. The two gene members were located in distinct clades with different characteristics, indicating their specific and diverse functions in non-pollinators. A previous study had reported that the PGRP gene family was different between pollinators and non-pollinators, and the PGRP-SA were absent in pollinators [17]. It is known that GNBP and PGRP-SA participate in the initiation of the Toll pathway, so the absence of both PGRP-SA and GNBP suggests a streamlined initiation style of the Toll pathway in pollinators. In CTL gene family, there were some lineage-specific clades in non-pollinators, such as clade 7, 12, and 13, which mainly existed or expanded in non-pollinators. CTLs participate in the immune recognition process, mediate hemocyte encapsulation and melanization, and maintain gut microbiome homeostasis [33,34,35]. Many CTLs act as pattern recognition receptors functioning in the immune response; nevertheless, some CTLs have unknown functions. Although the precise function of CTLs is yet to be revealed, the presence of specific genes and gene expansion events in non-pollinators implied that CTLs were more important in non-pollinators to resist pathogens. In addition, there was gene duplication of SCRBs in non-pollinators, suggesting that SCRBs may play important roles in resisting pathogens. These results suggested that recognition genes in the fig wasp immune system may be diverse, and the evolutionary patterns may be different between pollinators and non-pollinators. The diverse evolutionary patterns may be attributed to the differences in evolutionary history of the fig wasps associated with figs. Compared to non-pollinators, pollinators have spent much longer co-evolutionary with figs, and the fig ovaries provide a relatively safe and stable environment to pollinators, providing enough nutrients to pollinators and protecting them from pathogens [18]. Moreover, the lifestyles of pollinators and non-pollinators are different, since the non-pollinators spend much longer outside figs than pollinators, especially in the processes of mating and egg-laying; thus, their instances of exposure to pathogens are different.

The evolution of insect immune recognition receptors is closely related to their exposure to microorganisms [36]. Insects with more complex living environments are more likely to be exposed to a variety of pathogens and have higher requirements for the diversity of immune recognition genes [17,36]. Conversely, insects with fewer pathogens in their living environments have fewer immune recognition genes. For example, the house fly *M. domestica*, black soldier fly *H. illucens* and American cockroach *P. americana*, which live in dirty and messy environments, possess abundant immune recognition genes [9,11,12]. In contrast, the numbers of immune recognition genes of the body lice *Pediculus humanus* and tsetse fly *Glossina morsitans morsitans* are decreased, and it may be because they are fed on diets with fewer microbes [13,37]. Comparably, the differences of immune recognition genes between pollinators and non-pollinators also give us a hint that they have been subjected to different evolutionary pressures to adapt selective stresses, and needs for defense against external pathogens during their long evolutionary history.

## 5. Conclusions

Based on the genomes of 12 fig wasps with seven pollinators and five non-pollinators, we identified and analyzed putative PRRs. Comparative analysis revealed that pollinators had no GNBP, but non-pollinators had two gene members; phylogenetic analysis and characteristic analysis revealed that the two genes of non-pollinators were clustered into two clades, suggesting specific and diverse functions in non-pollinators. The CTL and SCRB gene families also showed differences between pollinators and non-pollinators; in non-pollinators, there were lineage-specific clades and gene expansion of CTLs and SCRBs. In conclusion, the comparative analysis results of PRRs suggested that some PRRs, such as GNBPs, CTLs, SCRBs, present different evolutionary patterns between pollinators and non-pollinators. Our results provide a molecular foundation for further functional studies of fig wasps, which would be beneficial to uncover the differences in immune recognition genes between pollinators and non-pollinators.

## Figures and Tables

**Table 1:** Gene number of pattern recognition receptors of the 12 fig wasp species and four reference species.

Group	Species	PGRP *	GNBP	CTL	SCRB	FREP	Galectin	TEP
Pollinators	*Kradibia gibbosae*	4	0	15	10	1	3	2
	*Ceratosolen fusciceps*	6	0	23	10	1	3	3
	*Ceratosolen solmsi*	6	0	17	10	1	3	3
	*Dolichoris vasculosae*	5	0	20	11	1	3	3
	*Eupristina koningsbergeri*	2	0	25	10	3	3	4
	*Platyscapa corneri*	4	0	24	12	1	3	3
	*Wiebesia pumilae*	5	0	19	10	1	3	3
Non-pollinators	*Apocrypta bakeri*	11	2	21	11	1	3	3
	*Philotrypesis tridentate*	10	2	27	11	1	3	3
	*Sycophaga agraensis*	6	2	15	11	1	3	3
	*Sycobia* sp.2	7	2	18	11	2	3	3
	*Sycophila* sp.2	13	2	26	11	1	3	4
Reference species	*Pteromalus puparum*	9	2	26	12	1	3	3
	*Nasonia vitripennis*	11	3	31	11	1	4	3
	*Apis mellifera*	4	2	12	10	1	3	3
	*Drosophila melanogaster*	13	3	34	13	13	6	6

PGRP, peptidoglycan recognition protein; GNBP, gram-negative bacteria-binding protein; CTL, C-type lectin; SCRB, scavenger receptor B; FREP, fibrinogen-related protein; TEP, thioester-containing protein. *, the gene numbers of PGRPs were from previous study [17].

**Table 2:** Statistical analysis of CTL genes for specific recognition of sugars in fig wasps.

Group	Species	Gene Name	Number	Binding Site	Sugar
Pollinators	*Ceratosolen fusciceps*	Cfus_CTL-5/6/7/11/20/22	6	QPD	galactose
		Cfus_CTL-8/12/17/18/19	5	EPN	mannose
	*Ceratosolen solmsi*	Csol_CTL-5/7/9/16	4	QPD	galactose
		Csol_CTL-15	1	EPN	mannose
	*Dolichoris vasculosae*	Dvas_CTL-1/4/10/11/14	5	QPD	galactose
		Dvas_CTL-3	1	EPN	mannose
	*Eupristina koningsbergeri*	Ekon_CTL-1/7/12/14/19/20/23	7	QPD	galactose
		Ekon_CTL-6/17	2	EPN	mannose
	*Kradibia gibbosae*	Kgib_CTL-2/8/11/12	4	QPD	galactose
		Kgib_CTL-6	1	EPN	mannose
	*Platyscapa corneri*	Pcor_CTL-2/11/17/19/21	5	QPD	galactose
		Pcor_CTL-6/7/14/15/18	5	EPN	mannose
	*Wiebesia pumilae*	Wpum_CTL-1/4/7/9/11/17	6	QPD	galactose
		Wpum_CTL-2/8	2	EPN	mannose
Non-pollinators	*Apocrypta bakeri*	Abak_CTL-4/5/6/7/8/10/11	7	QPD	galactose
		Abak_CTL-9	1	EPN	mannose
	*Philotrypesis tridentata*	Ptri_CTL-4/15/18(2)/19/22/24/25	7	QPD	galactose
		Ptri_CTL-7/10	2	EPN	mannose
	*Sycophaga agraensis*	Sagr_CTL-3/6/10/11	4	QPD	galactose
		Sagr_CTL-8/14/15	3	EPN	mannose
	*Sycobia* sp.2	Sbsp_CTL-3/13	2	QPD	galactose
		Sbsp_CTL-7/9/16	3	EPN	mannose
	*Sycophila* sp.2	Spsp_CTL-1/6/9/11/12/14/15/16	8	QPD	galactose
		Spsp_CTL-2/10/13/23	4	EPN	mannose

**Table 3:** Functional divergence analysis of three clades of galectin genes.

Clade	MFE *θ*	MLE *θ*	MFE Z Score	*p*-Value
clade 1/ clade 2	1.15 ± 0.226	1.03 ± 0.130	−6.266417	<0.01
clade 1/ clade 3	1.03 ± 0.212	0.9992 ± 0.125	−6.603005	<0.01
clade 2/ clade 3	0.99 ± 0.221	0.9992 ± 0.146	−5.908382	<0.01

**Table 4:** Functional divergence analysis of three clades of TEP genes.

Clade	MFE *θ*	MLE *θ*	MFE Z Score	*p*-Value
clade 1/clade 2	0.7826 ± 0.0956	0.6256 ± 0.0594	−10.343277	<0.01
clade 1/clade 3	0.7619 ± 0.0938	0.5832 ± 0.0717	−10.363998	<0.01
clade 2/clade 3	0.6499 ± 0.0966	0.596 ± 0.0757	−8.29982	<0.01

## Data Availability

The data presented in this study are openly available in NCBI with accession numbers of PRJNA277475, PRJNA641212 and PRJNA494992.

## Supplementary Materials

The following are available online at https://www.mdpi.com/article/10.3390/genes12121952/s1, Figure S1: The active sites of β-1,3-glucanase homolog domain of GNBPs. MAFFT software was used for GNBP amino acid sequence alignment. The asterisks indicated active sites of the key β-1,3-glucanase. The β-1,3-glucanase domain of AgGNBP of *Anopheles gambiae* had key active sites. Figure S2: Phylogenetic analysis of CTLs. The amino acid sequences of CTLs of 12 fig wasp species, *D. melanogaster* (Dm), *A. mellifera* (Am), *N. vitripennis* (Nv), and *P. puparum* (Pp) were used to construct the maximum likelihood tree, and the optimal model was JTT+R9. CTL-S, IML and CTL-X of CTL gene were represented by blue, red, and green respectively in the outer ring. The orthologs of fig wasps were represented by blue line clades. The numbers on the nodes represented clustered orthologs or specific gene clusters. The gene names of pollinators were marked with red, and those of non-pollinators were marked with blue. The groups of gene duplication in fig wasps were marked with red background and red dotted line branches. Figure S3: Genomic location and tandem repeat analysis of CTLs of fig wasp. Non-pollinators were filled with blue background and pollinators with pink background. The column represented the scaffold, and the colors of the scaffold indicated gene density (red indicated higher gene density, blue indicated lower gene density and white indicated no gene). The scaffolds were marked in yellow on the left, the CTL gene names were marked on the right, and the bar indicated the length (Mb). Tandem repeat genes were linked with red lines and large fragment replication was represented by “#”. Figure S4: Genomic location and tandem repeat analysis of SCRBs of fig wasps. The column represented the scaffold, which were marked in pink, and the scaffolds were marked in yellow on the left. The gene names were marked on the right, and the bar indicated the length (Mb). The number in parentheses represented the gene number of SCRB gene family of each species. Tandem repeat genes were linked with red lines. Figure S5: Phylogenetic relationships, conserved domains and motifs analysis of FREPs of 12 fig wasps. (A) The phylogenetic tree was transformed from Figure 5. (B) The distributions of conserved motif in FREP genes. Ten motifs were displayed in different colored boxes. (C) The domains of FREP genes. Each FREP gene contain a C-terminal fibrinogen domain. Table S1: The fig wasp species of pollinators and non-pollinators used in this study. Table S2: Sequence characteristics of pattern recognition receptors in fig wasps.
