# Supplementary material for: Genome-Wide Analysis of Gene Families of Pattern Recognition Receptors in Fig Wasps (Hymenoptera, Chalcidoidea)
Source: Genes (Basel). 2021 Dec 5;12(12):1952. doi: 10.3390/genes12121952 (PMC8702095; doi:10.3390/genes12121952)
Supplement: Supplementary file 1 [file genes-12-01952-s001.zip › Table S1.pdf]

**Table S1.** The fig wasp species of pollinators and non-pollinators used in this study.

| Group | Species | Abbreviation |
|---|---|---|
| Pollinators | *Ceratosolen solmsi* | Csol |
| | *Kradibia gibbosae* | Kgib |
| | *Wiebesia pumilae* | Wpum |
| | *Ceratosolen fusciceps* | Cfus |
| | *Dolichoris vasculosae* | Dvas |
| | *Eupristina koningsbergeri* | Ekon |
| | *Platyscapa corneri* | Pcor |
| Non-pollinators | *Sycobia* sp.2 | Sbsp |
| | *Sycophila* sp.2 | Spsp |
| | *Sycophaga agraensis* | Sagr |
| | *Apocrypta bakeri* | Abak |
| | *Philotrypesis tridentate* | Ptri |
