# Supplementary material for: Genome-Wide Analysis of Gene Families of Pattern Recognition Receptors in Fig Wasps (Hymenoptera, Chalcidoidea)
Source: Genes (Basel). 2021 Dec 5;12(12):1952. doi: 10.3390/genes12121952 (PMC8702095; doi:10.3390/genes12121952)
Supplement: Supplementary file 1 [file genes-12-01952-s001.zip › Table S2.pdf]

Table S2. Sequence characteristics of pattern recognition receptors in fig wasps.

Gene name	Gene family	Length (aa)	Isoele ctric point	Molecula r weight	Signal peptid e	Transmembrane Region
Abak_GNBP1	GNBP	429	9.15	49018.77	1-23	9-26
Abak_GNBP2	GNBP	355	6.67	40970.57	/	/
Sagr_GNBP1	GNBP	119	5.27	13580.53	1-22	5-27/96-113
Sagr_GNBP2	GNBP	386	6.87	44403.5	/	/
Ptri_GNBP1	GNBP	482	6.21	54958.73	/	/
Ptri_GNBP2	GNBP	358	5.65	40635.76	1-27	/
Sbsp_GNBP1	GNBP	464	5.78	53260.85	1-22	/
Sbsp_GNBP2	GNBP	339	6.54	38848.12	1-25	5-27
Spsp_GNBP1	GNBP	466	6.36	53653.11	1-19	/
Spsp_GNBP2	GNBP	359	6.11	40462.76	1-26	/
Abak_Gal-1	Galectin	336	7.17	39162.2	/	/
Abak_Gal-2	Galectin	1374	6.41	153174.36	/	/
Abak_Gal-3	Galectin	462	5.08	48728.83	/	/
Cfus_Gal-1	Galectin	1366	6.08	152564.4	/	/
Cfus_Gal-2	Galectin	431	8.95	49001.58	/	/
Cfus_Gal-3	Galectin	444	5.13	47918.12	/	/
Csol_Gal-1	Galectin	1172	5.79	130338.92	/	/
Csol_Gal-2	Galectin	265	6.6	30482.29	/	/
Csol_Gal-3	Galectin	423	5.38	45234.97	/	/
Dvas_Gal-1	Galectin	337	7.95	38812.02	/	/
Dvas_Gal-2	Galectin	454	5.31	48779.27	/	/
Dvas_Gal-3	Galectin	1399	7.87	158668.92	/	/
Ekon_Gal-1	Galectin	339	8.75	39276.46	/	/
Ekon_Gal-2	Galectin	1347	6.21	151100.73	/	/
Ekon_Gal-3	Galectin	445	5.56	48476.33	/	/
Kgib_Gal-1	Galectin	1342	6.58	149082.48	/	/
Kgib_Gal-2	Galectin	401	5.61	42792.47		
Kgib_Gal-3	Galectin	336	6.83	38566.55	/	/
Pcor_Gal-1	Galectin	1331	6.4	149215.28	/	/
Pcor_Gal-2	Galectin	450	5.33	48222.34	/	/
Pcor_Gal-3	Galectin	335	7.57	38835	/	/
Ptri_Gal-1	Galectin	357	8.03	41184.55	/	/
Ptri_Gal-2	Galectin	466	5.06	49257.5	/	/
Ptri_Gal-3	Galectin	1347	5.96	149994.2	/	/
Sagr_Gal-1	Galectin	308	6.46	35878.43	/	/
Sagr_Gal-2	Galectin	462	5.21	48706.76	/	/
Sagr_Gal-3	Galectin	1347	6.24	150786.98	/	/
Sbsp_Gal-1	Galectin	1344	6.4	150334.47	/	/

Gene name	Gene family	Length (aa)	Isoelectric point	Molecular weight	Signal peptide	Transmembrane Region
Sbsp_Gal-2	Galectin	481	5.05	49659.69	/	/
Sbsp_Gal-3	Galectin	369	6.48	42716.03	/	/
Spsp_Gal-1	Galectin	1347	5.98	150567.12	/	/
Spsp_Gal-2	Galectin	331	8.03	38503.54	/	/
Spsp_Gal-3	Galectin	436	5.25	45194.14	/	/
Wpum_Gal-1	Galectin	1346	6.34	150860.95	/	/
Wpum_Gal-2	Galectin	453	5.37	48432.21	/	/
Wpum_Gal-3	Galectin	336	7.12	38914.1	/	/
Abak_TEP-1	TEP	1880	6.06	207875.6	/	/
Abak_TEP-2	TEP	1706	6.47	195322.86	/	/
Abak_TEP-3	TEP	1453	5.52	163324.74	1-21	/
Cfus_TEP-1	TEP	1452	5.7	163898.91	1-22	/
Cfus_TEP-2	TEP	1661	6.03	190961.14	1-19	1628-1650
Cfus_TEP-3	TEP	1907	5.64	210918.52	/	/
Csol_TEP-1	TEP	1816	5.53	203041.58	1-18	/
Csol_TEP-2	TEP	1669	6.11	191557.67	1-20	1640-1658
Csol_TEP-3	TEP	1473	5.87	166005.19	1-22	7-29
Dvas_TEP-1	TEP	1436	6.03	165509.24	/	/
Dvas_TEP-2	TEP	1880	6.05	210123.36	/	5-24
Dvas_TEP-3	TEP	1487	5.77	167704.15	1-22	/
Ekon_TEP-1	TEP	1859	5.85	206252.15	1-19	/
Ekon_TEP-2	TEP	1317	5.67	147759.01	/	1288-1310
Ekon_TEP-3	TEP	1668	6.18	192074.67	1-25	1635-1657
Ekon_TEP-4	TEP	380	8.56	43341.95	/	/
Kgib_TEP-1	TEP	1658	6.28	190985.35	1-20	1628-1650
Kgib_TEP-2	TEP	1484	5.95	167849.16	1-22	/
Pcor_TEP-1	TEP	1855	5.88	206774.24	1-20	/
Pcor_TEP-2	TEP	1815	5.65	204002.64	1-22	7-29
Pcor_TEP-3	TEP	1664	6.05	191788.13	1-21	/
Ptri_TEP-1	TEP	1680	6.19	192869.17	1-19	/
Ptri_TEP-2	TEP	1484	5.78	166211.95	1-20	7-29
Ptri_TEP-3	TEP	1877	6.11	207327.05	1-18	/
Sagr_TEP-1	TEP	1477	5.83	166676.59	1-21	/
Sagr_TEP-2	TEP	1662	6.08	190738.9	1-19	1626-1648
Sagr_TEP-3	TEP	1862	5.8	207630.53	1-19	/
Sbsp_TEP-1	TEP	1681	6.18	193525.99	1-20	1648-1670
Sbsp_TEP-2	TEP	1498	6.5	169944.16	/	13-35
Sbsp_TEP-3	TEP	1918	5.73	210609.36	1-24	7-29
Spsp_TEP-1	TEP	1767	5.8	195268.23	1-19	/
Spsp_TEP-2	TEP	1647	6.17	189497.51	1-19	1624-1646

Gene name	Gene family	Length (aa)	Isoelectric point	Molecular weight	Signal peptide	Transmembrane Region
Spsp_TEP-3	TEP	1432	5.78	159976.2	1-19	/
Spsp_TEP-4	TEP	1605	5.91	184651.19	1-19	1581-1603
Wpum_TEP-1	TEP	1457	5.97	164208.1	1-22	/
Wpum_TEP-2	TEP	1883	5.89	208809.85	/	21-43
Wpum_TEP-3	TEP	1663	6.16	191295.46	1-20	/
Abak_CTL-1	CTL-S	173	5.35	19581.89	/	/
Abak_CTL-2	CTL-X	3535	5.22	386413.24	/	3392-3414
Abak_CTL-3	CTL-S	165	6.88	19001.77	/	/
Abak_CTL-4	CTL-S	206	5.45	22757.76	1-19	/
Abak_CTL-5	CTL-S	204	5.02	22798.69	1-22	/
Abak_CTL-6	CTL-S	200	5.4	22809.88	1-21	/
Abak_CTL-7	CTL-S	222	5.89	24622.93	1-19	/
Abak_CTL-8	CTL-S	217	6.88	24576.74	1-17	/
Abak_CTL-9	CTL-S	183	5.12	21038.65	1-23	/
Abak_CTL-10	CTL-S	295	8.8	33002.42	/	/
Abak_CTL-11	CTL-S	232	7.03	26602.05	1-19	/
Abak_CTL-12	CTL-X	1131	5.6	124565.98	/	1012-1034
Abak_CTL-13	CTL-S	403	5.27	46003.54	/	/
Abak_CTL-14	CTL-X	787	6.2	85968.52	1-21	752-774
Abak_CTL-15	CTL-S	193	5.08	21487.23	/	/
Abak_CTL-16	CTL-X	1074	8.4	126827.44	/	116-138/175-197/243-265/275-297/339-361/376-395/402-424/462-484/505-527/583-605/612-634
Abak_CTL-17	CTL-S	215	8.5	24276.69	1-21	/
Abak_CTL-18	CTL-X	900	5.03	103103.34	1-20	/
Abak_CTL-19	CTL-X	893	5.22	99054.94	/	/
Abak_CTL-20	CTL-S	283	8.4	31903.19	1-21	/
Abak_CTL-21	CTL-S	211	7.57	23518.92	/	7-26
Cfus_CTL-1	CTL-X	1311	6.23	143655.03	1-28	1192-1214
Cfus_CTL-2	CTL-X	742	5.28	82713.84	/	12-29
Cfus_CTL-3	CTL-X	830	7.76	91267.02	/	671-693
Cfus_CTL-4	CTL-X	3556	5.35	388592.24	1-23	3413-3435
Cfus_CTL-5	CTL-S	218	8.45	24815.75	1-18	/
Cfus_CTL-6	CTL-S	217	8.1	24788.01	1-17	/
Cfus_CTL-7	CTL-S	230	7.56	26350.86	1-17	/
Cfus_CTL-8	CTL-S	139	5.49	16593.45	/	/
Cfus_CTL-9	CTL-S	213	5.51	23321.24	1-19	/
Cfus_CTL-10	CTL-S	228	5.72	25322.9	/	12-34
Cfus_CTL-11	CTL-S	233	5.69	24925.29	1-17	/

Gene name	Gene family	Length (aa)	Isoelectric point	Molecular weight	Signal peptide	Transmembrane Region
Cfus_CTL-12	IML	534	4.99	59636.17	/	/
Cfus_CTL-13	CTL-S	213	4.39	23503.48	1-19	/
Cfus_CTL-14	CTL-S	213	4.48	23418.48	1-19	/
Cfus_CTL-15	IML	484	4.75	53598.14	1-19	/
Cfus_CTL-16	CTL-S	214	8.7	24386.91	1-20	/
Cfus_CTL-17	CTL-S	213	4.42	23048.83	1-19	/
Cfus_CTL-18	CTL-S	213	4.4	22949.76	1-19	/
Cfus_CTL-19	CTL-S	213	4.58	22941.87	1-19	/
Cfus_CTL-20	CTL-X	394	6.88	45015.53	1-26	7-26
Cfus_CTL-21	CTL-S	223	7.59	25943.69	1-16	/
Cfus_CTL-22	CTL-X	2090	10.1	220417.02	1-20	/
Cfus_CTL-23	CTL-S	254	8.5	28604.77	/	/
Csol_CTL-1	CTL-X	927	5.72	103539.74	/	/
Csol_CTL-2	CTL-S	156	9.5	18241.2	/	/
Csol_CTL-3	CTL-S	214	6.82	24986.45	/	/
Csol_CTL-4	CTL-X	663	6.61	72468.39	/	639-661
Csol_CTL-5	CTL-S	194	5.86	22529.24	/	/
Csol_CTL-6	CTL-S	122	6.48	13305.24	/	/
Csol_CTL-7	CTL-S	100	4.43	11020.27	/	/
Csol_CTL-8	IML	293	5.03	32842.98	/	/
Csol_CTL-9	CTL-S	142	5.75	15972.54	/	/
Csol_CTL-10	CTL-S	163	5.96	18735.25	/	/
Csol_CTL-11	CTL-S	163	8.51	17931.31	1-25	/
Csol_CTL-12	CTL-X	1092	5.51	119881.22	/	973-995
Csol_CTL-13	CTL-S	174	8.65	19383.04	/	/
Csol_CTL-14	CTL-X	2330	5.27	254588.72	/	/
Csol_CTL-15	CTL-S	134	5.06	15836.65	/	/
Csol_CTL-16	CTL-S	217	8.1	24832.11	1-17	/
Csol_CTL-17	CTL-S	329	10.23	38624.01	/	/
Dvas_CTL-1	CTL-S	217	8.1	25012.43	1-17	/
Dvas_CTL-2	CTL-X	1487	8.13	165250.46	/	1368-1390
Dvas_CTL-3	CTL-S	180	5.11	20772.21	1-20	/
Dvas_CTL-4	CTL-S	207	5.53	22096.21	/	/
Dvas_CTL-5	CTL-X	3625	5.3	396993.43	1-23	3482-3504
Dvas_CTL-6	CTL-S	289	8.31	33526.72	/	/
Dvas_CTL-7	CTL-S	835	5.66	97573.35	/	/
Dvas_CTL-8	CTL-S	692	5.13	78029.16	/	/
Dvas_CTL-9	IML	364	6.28	42986.23	/	/
Dvas_CTL-10	CTL-S	230	7.03	26469.93	1-17	/
Dvas_CTL-11	CTL-S	217	8.45	24773.72	1-17	/

Gene name	Gene family	Length (aa)	Isoelectric point	Molecular weight	Signal peptide	Transmembrane Region
Dvas_CTL-12	CTL-X	834	8.45	91651.39	/	673-695
Dvas_CTL-13	CTL-S	216	4.94	24680.97	/	/
Dvas_CTL-14	CTL-S	194	6.15	22444.97	/	/
Dvas_CTL-15	CTL-S	356	10.13	40800.5	1-21	202-224/300-322/332-349
Dvas_CTL-16	CTL-S	275	8.54	30690.78	/	/
Dvas_CTL-17	CTL-S	222	4.15	25133.06	1-21	/
Dvas_CTL-18	CTL-S	107	4.39	12317.85	/	/
Dvas_CTL-19	IML	472	8.24	54811.86	/	/
Dvas_CTL-20	CTL-X	1221	7.26	139562.73	/	/
Ekon_CTL-1	CTL-S	217	7.55	24821.08	1-16	/
Ekon_CTL-2	CTL-S	251	6.37	29170.83	1-15	/
Ekon_CTL-3	CTL-S	196	8.77	23002.84	1-18	/
Ekon_CTL-4	CTL-S	302	8.3	33961.73	1-18	/
Ekon_CTL-5	CTL-S	660	7.47	75448.39	/	/
Ekon_CTL-6	CTL-S	187	4.57	21189.65	1-20	/
Ekon_CTL-7	CTL-S	187	4.79	21234.94	1-20	/
Ekon_CTL-8	CTL-X	990	6.23	115358.55	/	/
Ekon_CTL-9	CTL-S	282	8.45	31891.52	1-19	/
Ekon_CTL-10	CTL-S	149	6.17	17170.13	1-20	/
Ekon_CTL-11	CTL-X	1086	5.53	119421.59	/	967-989
Ekon_CTL-12	CTL-S	230	7.03	26369.81	1-17	/
Ekon_CTL-13	CTL-S	183	4.14	20825.41	1-24	/
Ekon_CTL-14	CTL-S	184	4.48	21151.86	1-24	/
Ekon_CTL-15	CTL-X	792	8.09	87412.61	/	631-653
Ekon_CTL-16	CTL-S	252	6.94	29211.69	1-24	/
Ekon_CTL-17	CTL-S	120	4.51	14220.44	/	/
Ekon_CTL-18	CTL-X	501	7.76	59341.82	/	/
Ekon_CTL-19	CTL-S	217	8.45	24676.63	1-17	/
Ekon_CTL-20	CTL-S	141	5.44	16185.88	/	/
Ekon_CTL-21	CTL-S	218	5.55	24755.47	1-25	7-29
Ekon_CTL-22	CTL-S	253	8.07	28609.78	/	/
Ekon_CTL-23	CTL-S	218	5.57	23955.27	1-17	/
Ekon_CTL-24	CTL-S	243	5.49	28727.45	1-19	5-22
Ekon_CTL-25	CTL-X	1831	5.48	206078.35	/	/
Kgib_CTL-1	CTL-X	1373	5.53	151271.34	1-24	1005-1027
Kgib_CTL-2	CTL-S	217	8.11	24813.12	1-17	/
Kgib_CTL-3	CTL-S	213	8.34	24252.72	/	/
Kgib_CTL-4	CTL-X	795	6.08	93206.25	/	/
Kgib_CTL-5	CTL-S	251	9.19	27166.75	/	/
Kgib_CTL-6	CTL-S	180	5.22	20910.44	1-20	/

Gene name	Gene family	Length (aa)	Isoelectric point	Molecular weight	Signal peptide	Transmembrane Region
Kgib_CTL-7	CTL-S	523	6.62	61462.32	/	/
Kgib_CTL-8	CTL-S	226	5.95	24628.26	1-27	/
Kgib_CTL-9	CTL-X	905	6.62	99511.43	1-22	748-770
Kgib_CTL-10	CTL-S	189	9.28	22335.6	/	5-24
Kgib_CTL-11	CTL-S	217	8.45	24680.62	1-17	/
Kgib_CTL-12	CTL-S	230	7.6	26430.96	1-17	/
Kgib_CTL-13	CTL-S	412	5.03	46947.41	1-17	/
Kgib_CTL-14	CTL-X	748	5.48	82212.14	/	/
Kgib_CTL-15	CTL-X	3569	5.33	390527.05	/	3426-3448
Pcor_CTL-1	CTL-X	1138	6.06	124646.95	/	1019-1041
Pcor_CTL-2	CTL-S	151	4.61	17531.66	/	/
Pcor_CTL-3	CTL-S	274	4.95	30958.3	1-19	/
Pcor_CTL-4	CTL-S	429	9.58	48861.8	/	/
Pcor_CTL-5	CTL-S	198	4.56	21777.35	1-18	/
Pcor_CTL-6	CTL-S	197	4.45	22043.62	1-18	/
Pcor_CTL-7	CTL-S	153	5.24	17693.73	/	/
Pcor_CTL-8	CTL-S	200	8.45	22686.86	1-18	/
Pcor_CTL-9	CTL-S	149	4.88	16377.41	1-18	/
Pcor_CTL-10	CTL-S	270	8.82	29105.33	1-22	/
Pcor_CTL-11	CTL-S	198	5.59	22009.84	1-18	/
Pcor_CTL-12	CTL-S	198	5.3	21936.91	1-18	/
Pcor_CTL-13	CTL-S	262	9.77	30074.43	1-18	/
Pcor_CTL-14	CTL-S	197	4.92	21928.76	1-18	/
Pcor_CTL-15	CTL-S	198	4.78	22109.84	1-18	/
Pcor_CTL-16	CTL-S	914	4.47	101996.86	1-21	/
Pcor_CTL-17	CTL-S	258	8.14	29687.52	/	15-37/44-63
Pcor_CTL-18	CTL-S	221	6.09	25578.58	/	/
Pcor_CTL-19	CTL-S	230	7.03	26354.79	1-17	/
Pcor_CTL-20	CTL-X	3592	5.29	393541.37	1-23	3449-3471
Pcor_CTL-21	CTL-S	217	8.1	24994.34	1-17	/
Pcor_CTL-22	CTL-X	909	7.31	99294.97	1-24	748-770
Pcor_CTL-23	CTL-S	556	9.7	63339.58	/	/
Pcor_CTL-24	CTL-S	215	5.27	24179.56	1-21	/
Ptri_CTL-1	CTL-S	295	8.83	33272.08	/	/
Ptri_CTL-2	CTL-S	730	7.47	81131.04	1-23	/
Ptri_CTL-3	CTL-X	1064	5.63	117311.5	/	945-967
Ptri_CTL-4	CTL-S	221	5.66	24105.27	1-17	/
Ptri_CTL-5	CTL-S	239	6.3	26907.57	/	/
Ptri_CTL-6	CTL-X	879	5.58	101139.51	1-24	/
Ptri_CTL-7	CTL-S	206	5.96	23301.38	1-18	/

Gene name	Gene family	Length (aa)	Isoelectric point	Molecular weight	Signal peptide	Transmembrane Region
Ptri_CTL-8	CTL-S	211	6.23	23585.7	/	/
Ptri_CTL-9	CTL-X	1343	8.11	154910.18	/	29-51/61-80/127-149/164-186/244-266/276-298/327-344/1030-1052/1120-1142/1162-1184/1247-1269
Ptri_CTL-10	CTL-S	183	5.13	21030.65	1-23	/
Ptri_CTL-11	CTL-S	225	5.51	25513.7	/	/
Ptri_CTL-12	CTL-S	542	8.64	61437.32	/	/
Ptri_CTL-13	CTL-S	271	5.82	29554.66	1-22	/
Ptri_CTL-14	CTL-X	1071	8.87	115564.76	1-19	749-771
Ptri_CTL-15	CTL-S	216	7.55	24602.83	1-16	/
Ptri_CTL-16	CTL-S	347	8.31	38789.08	/	/
Ptri_CTL-17	CTL-X	3580	5.28	391751.54	1-23	3437-3459
Ptri_CTL-18	IML	410	5.36	46371.14	1-22	/
Ptri_CTL-19	CTL-S	207	4.86	22784.61	1-20	/
Ptri_CTL-20	CTL-S	213	6.08	24149.75	1-18	/
Ptri_CTL-21	CTL-S	203	9	22513.75	1-28	13-35
Ptri_CTL-22	CTL-S	210	7.02	23195.33	1-20	/
Ptri_CTL-23	CTL-S	110	8.99	13022.84	/	/
Ptri_CTL-24	CTL-S	218	8.45	24760.71	1-18	/
Ptri_CTL-25	CTL-S	232	7.03	26570.06	1-19	/
Ptri_CTL-26	CTL-X	2188	5.56	241749.79	/	/
Ptri_CTL-27	CTL-X	1224	6.33	139595.57	1-19	/
Sagr_CTL-1	CTL-S	107	6.56	12632.56	/	/
Sagr_CTL-2	CTL-S	149	6.7	17382.84	1-20	/
Sagr_CTL-3	CTL-S	186	6.7	21788.72	1-23	/
Sagr_CTL-4	CTL-S	295	8.86	33807.57	1-23	/
Sagr_CTL-5	CTL-S	349	9.31	40324.54	/	9-31
Sagr_CTL-6	CTL-S	217	8.46	24618.55	1-17	/
Sagr_CTL-7	CTL-X	1370	10.03	149746.18	/	/
Sagr_CTL-8	CTL-S	216	9.08	24876.92	1-24	/
Sagr_CTL-9	CTL-S	504	9.74	55834.74	1-25	/
Sagr_CTL-10	CTL-S	230	7.59	26307.77	1-17	/
Sagr_CTL-11	CTL-S	217	7.56	24753.97	1-17	/
Sagr_CTL-12	CTL-X	812	7.74	89192.37	/	662-684
Sagr_CTL-13	CTL-S	396	9.42	43203.28	1-21	/
Sagr_CTL-14	CTL-S	178	4.92	20671.28	1-22	/
Sagr_CTL-15	CTL-S	224	8.26	25500.2	1-26	/
Sbsp_CTL-1	CTL-S	197	6.98	22302.56	1-21	/
Sbsp_CTL-2	CTL-X	964	7.03	105065.2	/	794-816

Gene name	Gene family	Length (aa)	Isoelectric point	Molecular weight	Signal peptide	Transmembrane Region
Sbsp_CTL-3	CTL-S	219	8.46	24779.71	1-19	/
Sbsp_CTL-4	CTL-S	224	6.44	24457.81	/	5-27
Sbsp_CTL-5	CTL-S	226	6.74	24054.51	/	/
Sbsp_CTL-6	CTL-S	278	8.63	31346.06	1-21	/
Sbsp_CTL-7	CTL-S	199	7.97	22602.06	1-21	/
Sbsp_CTL-8	CTL-S	199	6.87	22368.7	1-21	/
Sbsp_CTL-9	CTL-S	164	5.91	18445.12	1-17	/
Sbsp_CTL-10	CTL-X	4427	5.34	487570.36	/	7-29/4284-4306
Sbsp_CTL-11	CTL-X	1119	5.7	123134.88	/	1000-1022
Sbsp_CTL-12	CTL-S	284	8.95	32985.66	/	/
Sbsp_CTL-13	CTL-S	219	7.55	24794.1	1-17	/
Sbsp_CTL-14	CTL-S	106	5.64	12201.82	/	/
Sbsp_CTL-15	CTL-S	301	9.42	32776.21	/	/
Sbsp_CTL-16	CTL-S	230	5.83	26474.61	1-20	/
Sbsp_CTL-17	CTL-X	2152	5.54	240281.68	1-24	/
Sbsp_CTL-18	CTL-X	1225	6.45	139721.9	/	/
Spsp_CTL-1	CTL-S	217	8.46	24681.6	1-17	/
Spsp_CTL-2	CTL-S	179	6.7	20854.75	1-20	/
Spsp_CTL-3	CTL-S	260	9.19	29296.64	1-21	/
Spsp_CTL-4	CTL-X	1116	5.82	122372.11	/	997-1019
Spsp_CTL-5	CTL-X	915	7.3	100517.74	1-28	762-784
Spsp_CTL-6	CTL-S	253	6.52	28791.56	1-16	/
Spsp_CTL-7	CTL-S	275	9.26	30798.99	1-18	240-262
Spsp_CTL-8	CTL-X	1236	5.95	141238.22	/	900-922/991-1013/1033-1054/1105-1127/1137-1156/1199-1218
Spsp_CTL-9	CTL-S	226	4.88	25654.07	1-18	/
Spsp_CTL-10	CTL-X	496	6.46	56585.81	/	41-63/73-95/129-151/190-212/267-289/299-321
Spsp_CTL-11	CTL-S	126	4.87	14687.56	/	/
Spsp_CTL-12	CTL-S	205	5.29	22750.53	1-18	/
Spsp_CTL-13	CTL-S	183	5.71	20738.79	/	/
Spsp_CTL-14	CTL-S	205	5.51	23221.33	1-19	/
Spsp_CTL-15	CTL-S	182	4.83	20150.56	/	/
Spsp_CTL-16	CTL-S	217	6.03	23646.88	1-17	/
Spsp_CTL-17	CTL-S	229	5.85	25253.29	/	/
Spsp_CTL-18	CTL-X	584	5.98	67051.41	/	/
Spsp_CTL-19	CTL-S	220	7.58	24935.7	/	/
Spsp_CTL-20	CTL-X	854	5.1	98011.21	1-22	/
Spsp_CTL-21	CTL-S	283	8.61	31935.19	1-19	/

Gene name	Gene family	Length (aa)	Isoelectric point	Molecular weight	Signal peptide	Transmembrane Region
Spsp_CTL-22	CTL-S	331	6.42	37826.05	/	/
Spsp_CTL-23	CTL-S	182	5.01	20888.47	1-22	/
Spsp_CTL-24	CTL-X	4175	5.47	456882.83	/	3439-3461
Spsp_CTL-25	CTL-S	348	10.15	40332.07	1-19	/
Spsp_CTL-26	CTL-X	2170	5.4	240471.94	1-20	/
Wpum_CTL-1	CTL-S	217	8.45	24787.75	1-17	/
Wpum_CTL-2	IML	363	9.02	41267.99	/	/
Wpum_CTL-3	CTL-S	281	8.57	32458.09	/	/
Wpum_CTL-4	CTL-S	207	6.59	24051.95	/	/
Wpum_CTL-5	CTL-S	219	6.64	25710.66	1-21	/
Wpum_CTL-6	CTL-X	728	5.25	81535.11	/	/
Wpum_CTL-7	CTL-S	217	8.1	25127.69	1-17	/
Wpum_CTL-8	CTL-S	180	5.02	20681.13	1-20	/
Wpum_CTL-9	CTL-S	188	5.26	21188.95	1-22	/
Wpum_CTL-10	CTL-S	183	6.21	20940.77	1-18	/
Wpum_CTL-11	CTL-S	188	5.26	21188.95	1-22	/
Wpum_CTL-12	CTL-X	1208	5.58	133537.51	/	1001-1023
Wpum_CTL-13	CTL-X	818	5.73	95697.61	/	/
Wpum_CTL-14	CTL-X	910	7.51	99456.3	1-20	7-29/748-770
Wpum_CTL-15	CTL-S	707	8.73	82304.84	/	/
Wpum_CTL-16	CTL-X	3566	5.3	390236.17	1-23	3423-3445
Wpum_CTL-17	CTL-S	219	5.61	23505.79	1-17	/
Wpum_CTL-18	CTL-S	213	8.79	24246.59	/	/
Wpum_CTL-19	CTL-X	552	6.52	61347.57	/	/
AbakSCR-B1	SCRB	503	7.86	56948.77	/	7-29/474-496
AbakSCR-B2	SCRB	590	7.6	66830.46	/	65-87/498-520
AbakSCR-B3	SCRB	538	7.07	61506.67	/	34-56/480-502
AbakSCR-B4	SCRB	522	8.28	56956.01	/	19-41/459-481
AbakSCR-B5	SCRB	579	6.69	66442.8	/	9-28/485-507
AbakSCR-B6	SCRB	563	5.83	63277.85	/	77-99/509-531
AbakSCR-B7	SCRB	523	7.06	58804.42	/	7-29/455-477
AbakSCR-B8	SCRB	539	8.4	61180.32	/	12-34/463-485
AbakSCR-B9	SCRB	542	5.38	61949.22	/	13-32/469-491
AbakSCR-B10	SCRB	604	5.92	69562.26	/	77-99/507-529
AbakSCR-B11	SCRB	531	8.3	59574.13	/	9-31
CfusSCR-B1	SCRB	525	9	59495.34	/	9-31/462-484
CfusSCR-B2	SCRB	554	6.4	63602.79	/	9-28/485-507
CfusSCR-B3	SCRB	562	7	63942.81	/	85-107/514-536
CfusSCR-B4	SCRB	629	7.9	72040.48	/	103-125/537-559
CfusSCR-B5	SCRB	537	5.72	60850.6	/	13-35/462-484

Gene name	Gene family	Length (aa)	Isoelectric point	Molecular weight	Signal peptide	Transmembrane Region
CfusSCR-B6	SCRB	588	8.44	66098.62	/	63-85/493-515
CfusSCR-B7	SCRB	545	8.17	60787.41	/	7-26/449-471
CfusSCR-B8	SCRB	544	9.16	61895.58	/	32-54
CfusSCR-B9	SCRB	525	8.25	58097.57	/	20-42/459-481
CfusSCR-B10	SCRB	499	8.31	56873.96	/	9-31/470-492
DvasSCR-B1	SCRB	473	8.82	54191.53	/	443-465
DvasSCR-B2	SCRB	588	8.25	66075.61	/	63-85
DvasSCR-B3	SCRB	537	5.98	61270.14	/	13-35/462-484
DvasSCR-B4	SCRB	524	8.25	59256.26	/	32-54/479-498
DvasSCR-B5	SCRB	522	8.28	57945.26	/	19-41/459-481
DvasSCR-B6	SCRB	668	8.89	76268.13	/	143-165/573-595
DvasSCR-B7	SCRB	486	8.2	54905.56	/	7-29/439-461
DvasSCR-B8	SCRB	555	5.94	63623.62	/	9-28/481-503
DvasSCR-B9	SCRB	490	8.82	55628.56	/	9-31/463-481
DvasSCR-B10	SCRB	572	8.67	65747.2	/	/
DvasSCR-B11	SCRB	519	6.16	59050.91	/	7-29/454-476
EkonSCR-B1	SCRB	617	7.48	69833.89	/	136-158/567-589
EkonSCR-B2	SCRB	522	7.58	59201.06	/	7-29/452-474
EkonSCR-B3	SCRB	522	8.8	59228.06	/	9-31/461-479
EkonSCR-B4	SCRB	550	5.14	62557.95	/	28-50/476-498
EkonSCR-B5	SCRB	551	5.82	63072.11	/	7-29/481-503
EkonSCR-B6	SCRB	735	6.76	83664.72	/	212-234/641-663
EkonSCR-B7	SCRB	522	8.28	57763.12	/	19-41/459-481
EkonSCR-B8	SCRB	523	8.6	59161.09	/	32-54/479-498
EkonSCR-B9	SCRB	487	7.12	55906.64	/	455-477
EkonSCR-B10	SCRB	767	8.86	86015.27	/	242-264/672-694
KgibSCR-B1	SCRB	503	6.29	57302.22	/	9-28/468-490
KgibSCR-B2	SCRB	554	5.78	63666.57	/	9-28/485-507
KgibSCR-B3	SCRB	550	8.86	62234.68	/	79-101/501-523
KgibSCR-B4	SCRB	583	5.53	66730.05	/	55-77/485-507
KgibSCR-B5	SCRB	525	7.98	58009.39	/	19-41/459-481
KgibSCR-B6	SCRB	526	8.24	59858.75	/	32-54/479-500
KgibSCR-B7	SCRB	537	5.45	60985.77	/	13-35/462-484
KgibSCR-B8	SCRB	588	7.13	66370.01	/	63-85/492-514
KgibSCR-B9	SCRB	531	8.66	60401.95	/	487-509
KgibSCR-B10	SCRB	522	7.94	59136.92	/	7-26/454-476
PcorSCR-B1	SCRB	590	5.63	67113.19	/	65-87/494-516
PcorSCR-B	SCRB	554	5.29	63373.41	/	33-55/481-503
PcorSCR-B3	SCRB	522	8.47	57718.27	/	19-41/459-481
PcorSCR-B4	SCRB	489	8.56	55633.51	/	7-29/439-461

Gene name	Gene family	Length (aa)	Isoelectric point	Molecular weight	Signal peptide	Transmembrane Region
PcorSCR-B5	SCRB	489	6.28	56006.29	/	429-451
PcorSCR-B6	SCRB	535	8.11	60749.21	/	9-31
PcorSCR-B7	SCRB	659	8.66	74542.71	/	595-617
PcorSCR-B8	SCRB	513	8.34	57959.59	/	7-29/458-475
PcorSCR-B9	SCRB	588	6.84	66178.62	/	63-85
PcorSCR-B10	SCRB	554	6.06	63271.25	/	9-28/485-507
PcorSCR-B11	SCRB	285	5.94	31984.53	/	240-259
PcorSCR-B12	SCRB	463	9.36	52208.35	/	32-54
PtriSCR-B1	SCRB	604	5.68	69517.88	/	72-94/504-526
PtriSCR-B2	SCRB	590	8.45	66469.02	/	63-85/496-518
PtriSCR-B3	SCRB	545	5.91	61327	/	42-64
PtriSCR-B4	SCRB	522	8.28	57028.09	/	19-41/459-481
PtriSCR-B5	SCRB	507	5.84	57572.36	/	7-29/477-499
PtriSCR-B6	SCRB	518	8.45	58660.57	/	30-52/463-485
PtriSCR-B7	SCRB	579	6.45	66429.72	/	9-28/485-507
PtriSCR-B8	SCRB	503	5.29	57645.22	/	426-448
PtriSCR-B9	SCRB	526	8.89	60564.13	/	13-32/464-486
PtriSCR-B10	SCRB	524	6.23	58760.42	/	7-29/453-475
PtriSCR-B11	SCRB	525	8.57	58860.62	/	9-31
SagrSCR-B1	SCRB	525	6.51	59318.91	/	32-54/477-499
SagrSCR-B2	SCRB	590	7.18	66413.82	/	65-87/498-520
SagrSCR-B3	SCRB	534	5.85	60910.85	/	45-67/480-502
SagrSCR-B4	SCRB	554	6.05	63835.03	/	9-28/484-506
SagrSCR-B5	SCRB	522	8.83	59415.37	/	10-32/431-449/464-483
SagrSCR-B6	SCRB	523	8.28	57204.21	/	19-41/459-481
SagrSCR-B7	SCRB	527	8.98	60039.82	/	9-31/463-485
SagrSCR-B8	SCRB	533	5.4	60839.65	/	13-30/466-488
SagrSCR-B9	SCRB	603	6.2	69559.51	/	77-99/509-531
SagrSCR-B10	SCRB	604	8.46	68335.28	/	83-105/534-556
SagrSCR-B11	SCRB	495	7.94	56586.47	/	7-29/469-491
SbspSCR-B1	SCRB	523	8.38	58386.22	/	10-32/430-448/463-482
SbspSCR-B2	SCRB	523	7.58	59369.84	/	7-26/453-475
SbspSCR-B3	SCRB	511	8.12	58316.11	/	21-43
SbspSCR-B4	SCRB	523	8.28	57964.2	/	19-41/459-481
SbspSCR-B5	SCRB	593	8.23	66692.25	/	63-85/496-518
SbspSCR-B6	SCRB	552	5.75	63619.57	/	9-28/485-507
SbspSCR-B7	SCRB	496	8.97	56689.48	/	7-29/442-464
SbspSCR-B8	SCRB	594	6.05	68191.91	/	65-87/497-519
SbspSCR-B9	SCRB	509	5.82	57847.83	/	13-35/434-456
SbspSCR-B10	SCRB	502	6.33	57462.91	/	472-494

Gene name	Gene family	Length (aa)	Isoelectric point	Molecular weight	Signal peptide	Transmembrane Region
SbspSCR-B11	SCRB	502	6.04	57304.15	/	16-38/463-485
SpspSCR-B1	SCRB	524	8.28	57586.69	/	19-41/460-482
SpspSCR-B2	SCRB	512	7.61	57916.33	/	20-42/465-487
SpspSCR-B3	SCRB	534	6.77	60675.41	/	13-30/457-479
SpspSCR-B4	SCRB	604	5.48	69299.75	/	78-100/509-531
SpspSCR-B5	SCRB	501	6.23	56826.07	/	12-34/470-492
SpspSCR-B6	SCRB	590	8.73	66528.22	/	65-87/498-520
SpspSCR-B7	SCRB	569	5.58	64232.24	/	85-107/517-539
SpspSCR-B8	SCRB	554	6.29	63415.57	/	9-31/485-507
SpspSCR-B9	SCRB	527	8.66	59667.99	/	9-31
SpspSCR-B10	SCRB	405	5.97	45128.45	/	337-359
SpspSCR-B11	SCRB	524	8.4	60288.77	/	9-31/464-486
WpumSCR-B1	SCRB	528	8.82	60125.07	/	9-31/466-484
WpumSCR-B2	SCRB	543	8.29	61448.42	/	7-26/476-498
WpumSCR-B3	SCRB	538	8.27	59886.67	/	19-41/459-481
WpumSCR-B4	SCRB	524	6.74	59484.24	/	31-53/479-498
WpumSCR-B5	SCRB	458	8.37	52346.2	/	430-452
WpumSCR-B6	SCRB	537	5.25	61081.78	/	13-35/462-484
WpumSCR-B7	SCRB	588	6.84	66173.69	/	63-85/493-515
WpumSCR-B8	SCRB	590	6.59	67675.28	/	65-87/494-516
WpumSCR-B9	SCRB	557	8.86	63220.31	/	82-104/510-532
WpumSCR-B10	SCRB	555	6.81	63669.74	/	9-28/485-507
CsolSCR-B1	SCRB	582	5.82	66549.74	/	56-78/486-508
CsolSCR-B2	SCRB	436	5.42	48932.3	/	/
CsolSCR-B3	SCRB	401	6.14	45955.49	/	332-354
CsolSCR-B4	SCRB	522	6.14	59259.97	/	7-26/454-476
CsolSCR-B5	SCRB	302	6.39	34158.74	/	7-29
CsolSCR-B6	SCRB	455	6.77	51894.47	/	373-395
CsolSCR-B7	SCRB	407	5.73	46518.81	/	332-354
CsolSCR-B8	SCRB	224	7.64	24024.84	/	158-180
CsolSCR-B9	SCRB	411	6.92	46755.08	/	32-54/366-385
CsolSCR-B10	SCRB	566	4.86	65019.39	/	514-536
Abak_FREP	FREP	607	7.59	67185.74	1-20	/
Cfus_FREP	FREP	642	6.66	72106.06	1-22	/
Csol_FREP	FREP	642	6.62	71982.13	1-21	/
Dvas_FREP-1	FREP	650	6.66	73174.26	1-21	/
Ekon_FREP-1	FREP	652	6.24	73837.92	1-20	/
Ekon_FREP-2	FREP	259	5.88	29805.62	1-16	/
Ekon_FREP-3	FREP	183	10.61	21490.7	/	/
Kgib_FREP-1	FREP	724	9.64	83613.56	/	/

Gene name	Gene family	Length (aa)	Isoelectric point	Molecular weight	Signal peptide	Transmembrane Region
Pcor_FREP-1	FREP	654	6.27	73462.62	1-22	/
Ptri_FREP	FREP	618	8.87	67605.31	1-21	/
Sagr_FREP-1	FREP	648	7.6	71785.8	1-30	12-34
Sbsp_FREP-1	FREP	597	6.32	66479.78	/	/
Sbsp_FREP-2	FREP	201	5.15	22449.92	/	/
Spsp_FREP	FREP	647	6.35	72204.93	1-21	/
Wpum_FREP	FREP	652	6.05	73399.63	1-21	/
