# Supplementary figures and images for: Genome-Wide Analysis of Gene Families of Pattern Recognition Receptors in Fig Wasps (Hymenoptera, Chalcidoidea)
Source: Genes (Basel). 2021 Dec 5;12(12):1952. doi: 10.3390/genes12121952 (PMC8702095; doi:10.3390/genes12121952)

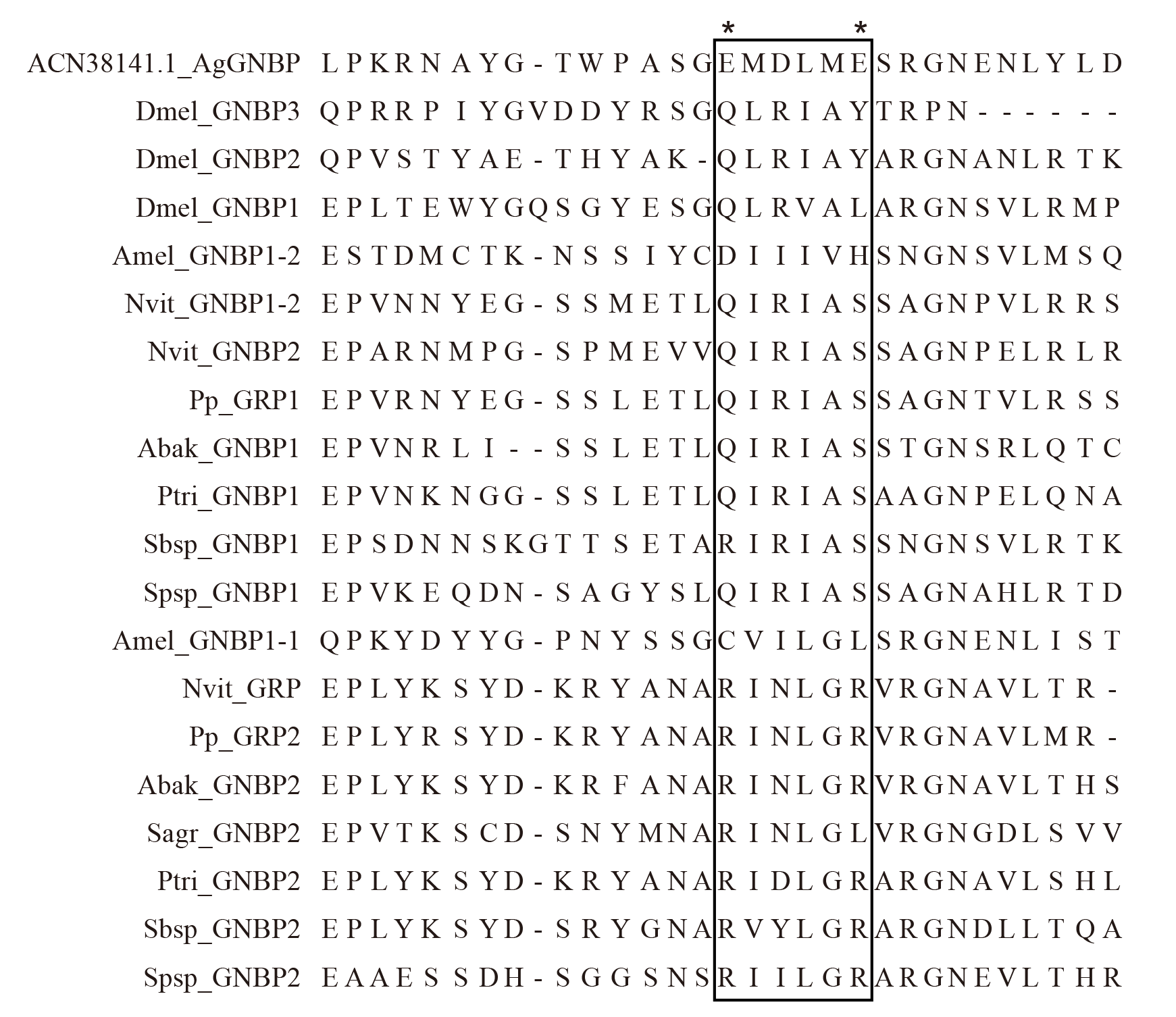

Supplement: Supplementary file 1 [file genes-12-01952-s001.zip › Figure S1.tif]

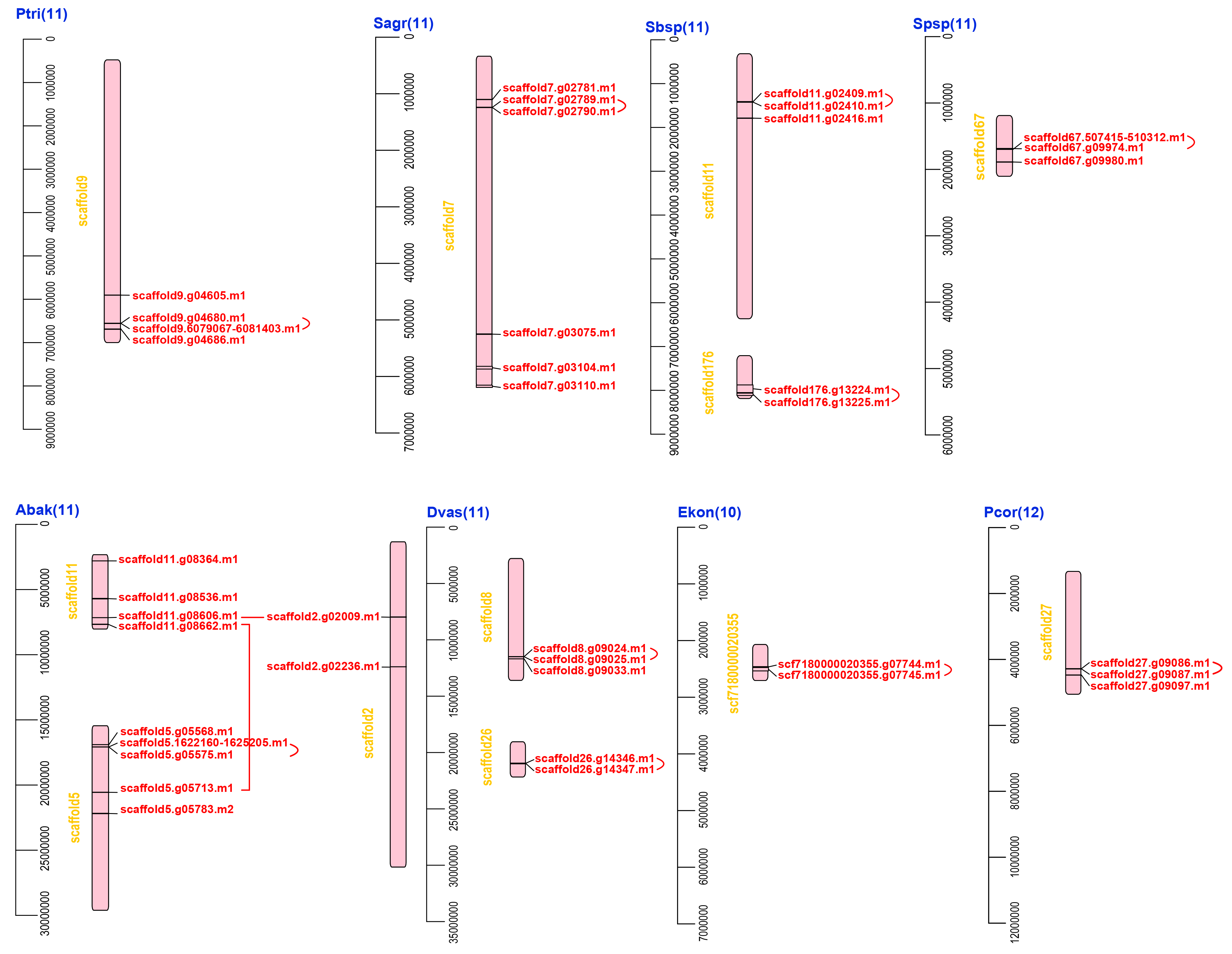

Supplement: Supplementary file 1 [file genes-12-01952-s001.zip › Figure S4.tif]

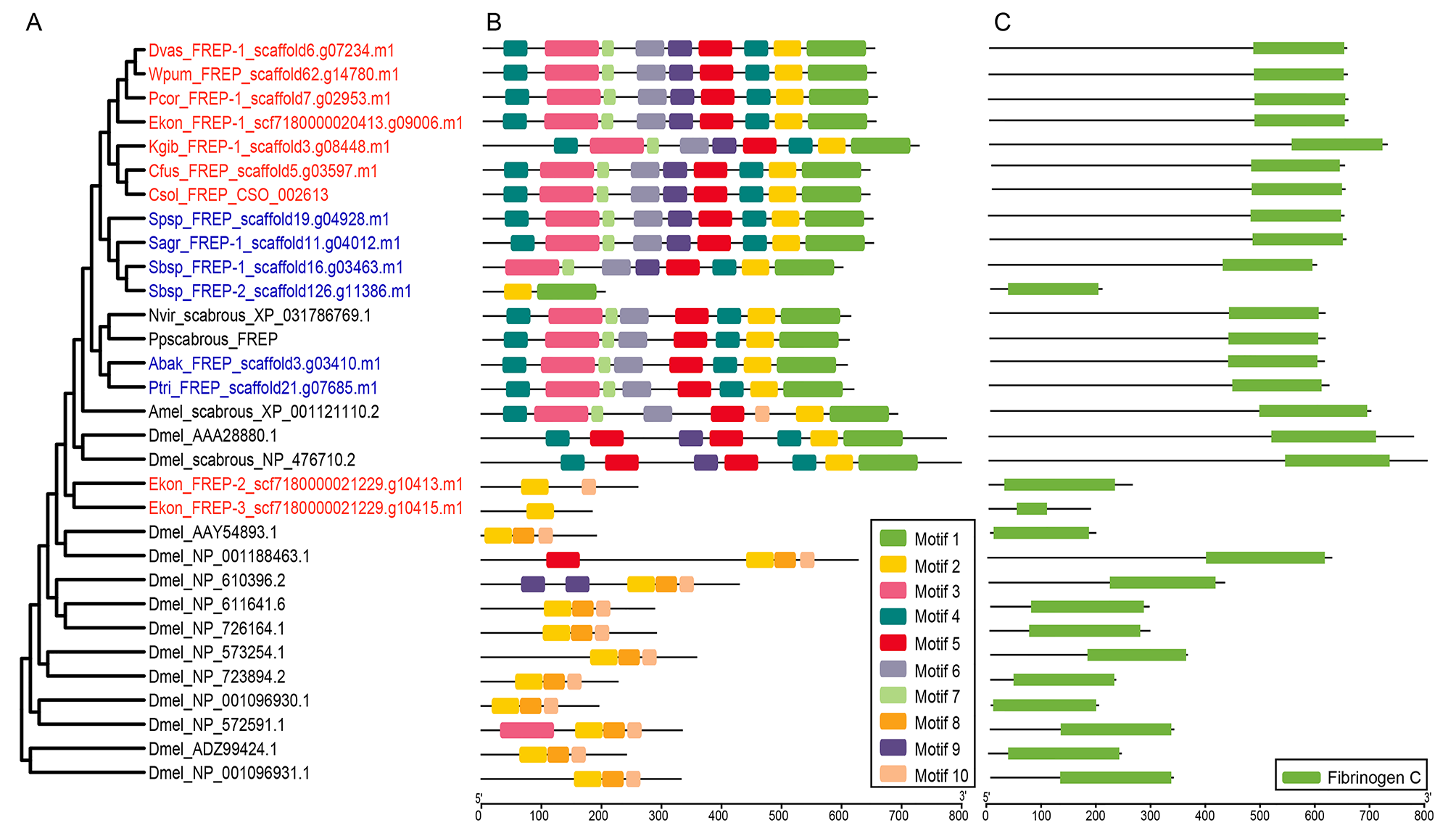

Supplement: Supplementary file 1 [file genes-12-01952-s001.zip › Figure S5.tif]
